# What’s in a name field?

**DOI:** 10.23889/ijpds.v9i5.2694

**Published:** 2024-09-18

**Authors:** Haley Golding, Katie Churchill, Charissa Levy, Lesley Plumptre

**Affiliations:** 1 ICES; 2 Rehabilitative Care Alliance

## Objective

To describe clinical pathways and use of the bundled care funding model, where a patient’s treatment and payment is assigned to a group of providers, among individuals who underwent a total joint arthroplasty (TJA) in Ontario. The request for this work was submitted by the Rehabilitative Care Alliance through the Applied Health Research Question (AHRQ) program.

## Approach

Adults with TJA from April 1st 2018 to March 31st 2022 were included. Demographics, surgical characteristics, inclusion in bundled care, and use of health services were defined using administrative health data. Results were stratified by location of surgery (bilateral or unilateral hip, knee, or shoulder TJA).

## Results

Among 152,135 individuals with TJA during the study period, 71,996 individuals (47.3%) received services through a bundled care approach. Rate of bundled care use was highest among individuals with unilateral knee TJA (52.5% in bundled care), and the lowest among individuals with shoulder TJA (13.4% in bundled care). In fiscal year 2018, 33% of TJA patients used bundled care, in fiscal year 2019, over 50% of TJA patients used bundled care and 55% of TJA patients used bundled care by 2021. Most individuals used bundled care following an inpatient surgery (88.9% of individuals following a unilateral knee TJA) as opposed to a same day surgery (11.1% with a unilateral knee TJA).

## Conclusion

The purpose of bundled care service delivery is to provide integrated healthcare delivery and therefore improved patient experience and outcomes. Understanding bundled care use may improve healthcare planning and assessment of health services.

